# Supplementary material for: Unpacking sources of transmission in HIV prevention trials with deep-sequence pathogen data
Source: Nat Commun. 2026 Mar 14;17:3935. doi: 10.1038/s41467-026-70203-x (PMC13128811; doi:10.1038/s41467-026-70203-x)
Supplement: Supplementary file 1 — Supplementary Information [file 41467_2026_70203_MOESM1_ESM.pdf]

Unpacking sources of transmission in HIV prevention trials with deep-sequence pathogen data

Supplementary Note S1 Statistical approach to estimate the relative contribution of various sources of infection in cluster-randomized trials of infectious disease prevention

S1.1 Population-based molecular source attribution model

In cluster-randomized trials of HIV prevention, where the randomization unit is communities, new infections can arise from individuals: in the same community, in different communities randomized to the same trial arm, in different communities randomized to the opposite trial arm and in non-trial communities. The ultimate aim of the analysis is to estimate the proportions of transmissions in trial communities that are attributable to each of the above-mentioned sources of infection.

To reach that aim, we posit a model of the probability $\pi_{ij}(t)$ that two HIV cases in persons of opposite-sex in community *i* and community *j* (where *i* and *j* can be different or the same community) at time *t* form a transmission pair, meaning that their viruses are genetically-linked, with transmission direction from *j* (source) to *i* (recipient). Note that $\pi_{ij}(t)$ is the probability that if these viruses were deep-sequenced, they would show such a linkage, and does not account for the probability that they are sampled. To estimate the model, we assume random sampling of persons with HIV from the population of persons of their sex with HIV in their respective communities. The model thus defines the probability of a linked pair, on

average, as the ratio of the number of linked opposite-sex pairs with a recipient in i and a source in j at time t identified in the data, $x_{ij}^{pairs}(t)$, and the maximum possible number of such linked pairs based on the number of deep sequences obtained from each sex in the two communities, $n_{ij}^{seq}(t)$, a known feature of the design:

$$\pi_{ij}(t) = \frac{E(x_{ij}^{pairs}(t))}{n_{ij}^{seq}(t)} . \qquad (1a)$$

To estimate $\pi_{ij}(t)$ we fit a negative binomial regression $x_{ij}^{pairs}(t) \sim Negbin\left(E(x_{ij}^{pairs}(t)), \theta\right)$, where

$$\ln(\frac{E(x_{ij}^{pairs}(t))}{n_{ij}^{seq}(t)}) = \beta_0 + \beta_1 c_j + \beta_2 s_{ij} + \beta_3 d_{ij}. \qquad (1b)$$

Here $c_j = 1$ if the source community is a nonintervention community, that is a control community or not in the study (non-trial community) and 0 otherwise, $s_{ij} = 1$ if i and j are the same community (within-community transmission) and 0 otherwise (between-community transmission), and d_{ij} is the distance in kilometers separating communities i and j.

Equivalently, $\ln\left(E(x_{ij}^{pairs}(t))\right) = \ln\left(n_{ij}^{seq}(t)\right) + \beta_0 + \beta_1 c_j + \beta_2 s_{ij} + \beta_3 d_{ij}. \qquad (1c)$

We describe $n_{ij}^{seq}(t)$ at baseline as $n_{ij}^{seq}(t_{baseline}) = n_{m_j}^{seq}(t_{baseline}) * n_{f_i}^{seq}(t_{baseline}) + n_{f_j}^{seq}(t_{baseline}) * n_{m_i}^{seq}(t_{baseline})$ and post-baseline as $n_{ij}^{seq}\left(t_{post-baseline}\right) = n_{m_j}^{seq}\left(t_{baseline} + t_{post-baseline}\right) * n_{f_i}^{seq}\left(t_{post-baseline}\right) + n_{f_j}^{seq}\left(t_{baseline} + t_{post-baseline}\right) * n_{m_i}^{seq}\left(t_{post-baseline}\right)$ where n_f^{seq} and n_m^{seq} represent sequenced females and males. The idea here is that persons who are HIV+ at baseline could not have been infected by people infected only post-baseline, but persons post-baseline could have been infected by persons who had HIV at either time

point. The input data for this regression are the 900 = 30 x 30 counts of transmissions between ordered pairs of the 30 trial communities (Supplementary Figure 1) comprising 51 transmissions where the recipient's genome was sampled at baseline and 31 are transmissions where the recipient's genome was sampled post-baseline. Note that $\pi_{ij}(t)$ simply represents the risk of transmission to recipients in community *i* from sources in community *j* at time *t* and that $\pi_{ij}(t)$ is the quantity we estimate in Table 1 and Figure 1. Also, because of data limitations models in Table 1 and Figure 1 were fit using 870 = 30 x 30 - 30 counts of transmissions (instead of 900). Specifically, there were no individuals sampled from Digawana intervention community during the post-baseline period whose HIV-1 virus was successfully deep-sequenced and met inclusion criteria for phylogenetic analysis.

Extrapolating beyond the trial communities for potential sources of transmission but focusing on the trial communities as recipients, we then use the estimated model for $\pi_{ij}(t)$, along with drive distances separating each of the 488 census communities in Botswana and the trial communities, to estimate the probability that a case in each of the trial communities is linked to a source in each of the census communities in the country, grouped as the same community, another trial community (control or intervention separately), or a non-trial community, which is treated as if it were a control community. Afterwards, we combined these estimates of the probability of linkage with estimates of NH_j the number of individuals living with HIV in each census community j in Botswana where *N* is population-size and *H* is HIV prevalence, and estimates of NH_i the number of individuals living with HIV in each trial community *i*, to

estimate the total number of transmissions to each trial community and the proportion of transmissions attributable to each of the above-mentioned probable source groups.

We estimate the number of transmissions to recipients in community *i* that occurred from individuals (probable sources) in community *j* as

$$z_{ij}(t) = \pi_{ij} Y_{ij}(t), \qquad (2a)$$

where, as indicated earlier, the probability $\pi_{ij}(t)$ denotes the risk of transmission to recipients in community *i* from sources in community *j* at time *t* or more formally the expected probability of viral-linkage between deep-sequenced HIV viruses of individuals randomly sampled from their respective communities *i* and *j*. In a scenario where the source community *j* is a community outside the trial area (non-trial community) $\pi_{ij}(t)$ denotes the risk of transmission to *i* from *j* at time *t* had the source cases been sequenced. $Y_{ij}(t)$ represents the maximum possible number of linked pairs of opposite-sex in the population that involve a probable source in community *j* and a recipient in community *i* at time *t*. We estimate $Y_{ij}(t)$ as $Y_{ij}(t) = NH_{m_j} * NH_{f_i} + NH_{f_j} * NH_{m_i}$ where NH_{m_j} and NH_{f_j} are the number of males and females respectively with HIV in source community *j* and NH_{m_i} and NH_{f_i} are the number of males and females respectively with HIV in recipient community *i*. A summary listing of the data, quantities and parameters estimated in the model is provided below:

Summary of quantities and model parameters

data and quantities

N_{f_i}, N_{f_j} female population-size of recipient community *i* and source community *j respectively*

N_{m_i}, N_{m_j} male population-size of recipient community *i* and source community *j respectively*

H_{f_i}, H_{f_j} female HIV prevalence of recipient community *i* and source community *j respectively*

H_{m_i}, H_{m_j} male HIV prevalence of recipient community *i* and source community *j respectively*

$NH_{f_i} = N_{f_i} * H_{f_i},\ NH_{f_j} = N_{f_j} * H_{f_j}$ number of females with HIV in recipient community *i* and source community *j respectively*

$NH_{m_i} = N_{m_i} * H_{m_i},\ NH_{m_j} = N_{m_j} * H_{m_j}$ number of males with HIV in recipient community *i* and source community *j respectively*

$n_{f_i}^{seq}(t), n_{f_j}^{seq}(t)$ number of females randomly sampled from recipient community *i* and source community *j*, respectively at time *t* whose viral whole genomes were successfully deep-sequenced

$n_{m_i}^{seq}(t), n_{m_j}^{seq}(t)$ number of males randomly sampled from recipient community *i* and source community *j*, respectively at time *t* whose viral whole genomes were successfully deep-sequenced

$n_{ij}^{seq}(t) = n_{m_j}^{seq}(t) * n_{f_i}^{seq}(t) + n_{f_j}^{seq}(t) * n_{m_i}^{seq}(t)$ maximum number of distinct possible

(opposite-sex) transmission pairs at time *t* between individuals randomly sampled from recipient community *i* and source community *j* whose viral whole genomes were successfully deep-sequenced

$Y_{ij}(t) = NH_{m_j} * NH_{f_i} + NH_{f_j} * NH_{m_i}$ maximum number of distinct possible (opposite-sex) transmission pairs in the population that involve a source in community *j* and a recipient in community *i*

$x_{ij}^{pairs}(t)$ number of transmission pairs identified from the deep-sequenced HIV virus of individuals randomly sampled from recipient community *i* and source community *j* at time *t*

c_j source community *j* is a nonintervention community, that is, a control community or non-trial community (yes = 1, no = 0)

s_{ij} source community and recipient community are the same, that is, same community transmission (yes = 1, no = 0)

d_{ij} distance in kilometers separating the source community *j* and recipient community *i*

and estimated parameters

$\pi_{ij}(t) = \frac{E(x_{ij}^{pairs}(t))}{n_{ij}^{seq}(t)}$ risk of HIV transmission to recipients in community *i* from sources in community *j* at time *t*

θ overdispersion parameter

$\beta_0, \beta_1, \beta_2, \beta_3$ fixed effects regression parameters.

To aid clarity, we now provide an example in which we estimate the proportion of transmissions to community *i* (an intervention community in the trial), at time *t*, that occurred from probable sources in the four groups of interest: same community, other intervention communities, control communities and non-trial communities. We describe the total estimated number of transmissions to recipients in community *i* at time *t* as the vector

$$\hat{z}_i(t) = (\hat{z}_{ia}(t),\ \hat{z}_{ib}(t),\ \hat{z}_{ic}(t),\ \hat{z}_{id}(t)), \tag{2b}$$

where $\hat{z}_{ia}(t)$ is the estimated number of transmissions from individuals within the same community, that is, community *i*, $\hat{z}_{ib}(t)$ is the estimated number of transmissions from individuals in other communities that are in the same trial arm as community *i*, $\hat{z}_{ic}(t)$ is the estimated number of transmissions from individuals in communities that are in the opposite trial arm to community *i* and $\hat{z}_{id}(t)$ is the estimated number of transmissions from individuals in non-trial communities. Then we estimate the proportions of transmissions to recipients in community *i* from individuals (sources of infection) in the four community groups of interest as:

$$\hat{\zeta}_i^{\,same\ community}(t) = \frac{\hat{z}_{ia}(t)}{(\hat{z}_{ia}(t) + \hat{z}_{ib}(t) + \hat{z}_{ic}(t) + \hat{z}_{id}(t))}, \tag{3a}$$

$$\hat{\zeta}_i^{\,same\ trial\ arm}(t) = \frac{\hat{z}_{ib}(t)}{(\hat{z}_{ia}(t) + \hat{z}_{ib}(t) + \hat{z}_{ic}(t) + \hat{z}_{id}(t))}, \tag{3b}$$

$$\hat{\zeta}_i^{\,opposite\ trial\ arm}(t) = \frac{\hat{z}_{ic}(t)}{(\hat{z}_{ia}(t) + \hat{z}_{ib}(t) + \hat{z}_{ic}(t) + \hat{z}_{id}(t))}, \tag{3c}$$

$$\hat{\zeta}_i^{\,non-trial\ community}(t) = \frac{\hat{z}_{id}(t)}{(\hat{z}_{ia}(t) + \hat{z}_{ib}(t) + \hat{z}_{ic}(t) + \hat{z}_{id}(t))}\ . \tag{3d}$$

For the summary measure of the intervention communities shown in Figure 3 we define a set of $\hat{\bar{\zeta}}_I$ (the same principle will apply for $\hat{\bar{\zeta}}_C$ for the control communities). Consider that *n* communities in the trial are randomized to receive the intervention, *I* := {1,2,…, n}. Then we estimate $\hat{\bar{\zeta}}^{same\ community}(t)$ the mean of the proportion of transmissions to recipients in intervention communities from individuals in the same community as

$$\hat{\bar{\zeta}}_I^{\,same\ community}(t) = \frac{\sum_{i \in I} \hat{z}_{ia}(t)}{\sum_{i \in I} \hat{z}_i(t)}\ . \tag{4}$$

where $\sum_{i \in I} \hat{z}_{ia}(t)$ is the total estimated transmissions at time *t* that occurred to trial communities randomized to receive the intervention, from sources in the same community, and $\sum_{i \in I} \hat{z}_i(t)$ is the total estimated transmissions at time *t* that occurred to intervention communities from probable sources in the same community, other intervention communities, control communities and non-trial communities. The same principle follows for the three other sources of infection: same trial arm, opposite trial arm and non-trial community; wherein the numerator for the mean estimate is the sum of the numerators of the individual community estimates and similarly, the denominator for the mean estimate is the sum of the denominators of the individual community estimates (Figures 2 and 3). Note that $\hat{\bar{\zeta}}^{same\ community}(t)$

$$+ \hat{\hat{\zeta}}^{same\ trial\ arm}(t) + \hat{\hat{\zeta}}^{opposite\ trial\ arm}(t) + \hat{\hat{\zeta}}^{non-trial\ community}(t) = 1.$$

Model assumptions

The central assumptions of this model are that the transmission pairs identified between samples from communities *i* and *j* are independent, and that transmission patterns in communities randomized to the control arm of the trial are representative of those found in non-trial communities. The simplifying assumption of independence is appropriate when identified transmission events mostly comprise small two-person clusters as found in the BCPP data and could be less-suited for large clusters typical of super spreader events. Furthermore, we assume that population-size and HIV prevalence of communities *i* and *j* are known, and that the HIV prevalence in administrative districts is representative of that in communities. We acknowledge that community-level HIV prevalence estimates can be obtained directly with the methods of [1] and reserve such computation for future study.

S1.2 Application to BCPP study data

We applied the population-based molecular source attribution model in section S1.1 to estimate the relative contribution of sources of infection (inside and outside the trial area) to transmissions that occurred to recipients in BCPP trial communities. First, as described in equations 1a to 1c we estimated $\pi_{ij}(t)$, the risk of transmission between ordered pairs of the 30 trial communities in BCPP (see Table 1, Figure 1 and methods *Deep-sequence phylogenetics data*). Estimates were obtained using parametric maximum likelihood estimation with the nbreg module in Stata 13.1 and the glm.nb function in the MASS package v7.3-54 in R v4.1.2 [2]. Next, we used the post-baseline model of the risk of transmission between ordered pairs of communities in the BCPP trial to predict the risk of transmission to trial communities (intervention communities and control communities) from the 488 communities that participated in the 2011 Botswana population and housing census. Note that the 488 census communities included all 30 communities in the BCPP trial (see methods *Pairwise drive distance data*). The risk prediction dataset comprised (14,152 = 488 x 29) distinct observations that each contained five pieces of information. These were the same first four pieces of information as those in the input dataset to estimate the risk of transmission between BCPP trial communities, namely: 1) ordered community pair, that is, source community and recipient community, 2) non-intervention community status c_j, 3) same community transmission status s_{ij}, 4) drive distance in kilometers separating the source community and recipient community d_{ij} (see methods *Pairwise drive distance data*) and for the fifth piece of information, we set the natural-log of the maximum number of distinct possible (opposite-sex) transmission pairs between

individuals randomly sampled from the source community and recipient community during the relevant time period whose viral genomes were successfully sequenced to zero, that is, $\ln(n_{ij}^{seq}(t)) = 0$. We set $\ln(n_{ij}^{seq}(t)) = 0$ to predict the risk of transmission (expected probability of viral linkage) instead of the expected transmission counts. In each ordered community pair, we used the origin community in an origin-destination pairing as a surrogate for the source community and the destination community as a surrogate the recipient community. Predictions were made with the predict function in the stats package in R v4.1.2. Then as described in equation 2a we estimated the maximum possible number of linked pairs of opposite-sex in the population that involve a probable source in the 488 census communities and a recipient in a trial community post-baseline, $Y_{ij}(t_{post-baseline})$ (see methods *Population-size and HIV prevalence estimates*). From there, we estimated the number of transmissions between each of the (14,152 = 488 x 29) ordered pairs of communities as $z_{ij}(t_{post-baseline}) = \pi_{ij} Y_{ij}(t_{post-baseline})$.

Alternative models

We considered alternative versions of the post-baseline model for the risk of transmission between ordered pairs of communities in the BCPP trial that used transforms of the pairwise drive distance separating communities (Supplementary Table 1). The linear model shown in supplementary Table 1 and described in equations 1a through 1c was selected as the best model based on Aikaike information criterion (AIC) and parsimony. We noted that sampling of trial participants through clinics in the BCPP trial was only done in intervention communities

but not in control communities resulting in an asymmetry between the intervention and control arms of the trial. Therefore, we modeled the risk of transmission between ordered pairs of communities in the BCPP trial excluding transmission pairs where both individuals were sampled at a clinic in an intervention community during the same trial period, that is, both individuals sampled at baseline or post-baseline (Supplementary Table 2). We found similar patterns to those observed in the baseline and post-baseline models in Table 1. The negative-binomial regression models used to estimate the risk of transmission between ordered pairs of communities in the BCPP trial were fit with parametric maximum likelihood estimation with the nbreg module in Stata 13.1 and the glm.nb function in the MASS package v7.3-54 in R v4.1.2 [2].

Model diagnostics

We performed several diagnostics to assess the fit of the post-baseline model in Table 1 to the directed transmission pairs identified between ordered pairs of communities in the BCPP trial. First, we assessed if the model converged to the maximum likelihood of the data using a likelihood grid search wherein the mean number of transmission pair counts predicted by the post-baseline model in Table 1 was adjusted upwards and downwards by 10% and 20%. We found that adjusting the predicted number of counts upwards or downwards did not improve the log-likelihood suggesting that the model had converged on the maximum likelihood of the data. Second, we compared the number of transmission pairs identified between ordered pairs of communities in the BCPP trial with those that would be expected under the post-baseline model in Table 1. There was little evidence to suggest that the observed counts differed

substantially from those expected under the model (Fisher exact $P = 0.792$) (Supplementary Table 3). Third, we also used a simulation-based approach to compare the distribution of observed quantile residuals with that expected under the post-baseline model in Table 1 and found little appreciable difference between the observed and expected distributions (Supplementary Figure 3). The simulation-based approach was performed using the DHARMa package v0.4.6 in R v4.1.2.

Confidence intervals

We used an empirical bootstrap approach to compute 95% confidence intervals for the estimated proportions of transmissions attributable to different sources of infection wherein each bootstrap procedure was performed with 1,000 replicates. The lower and upper bounds of the confidence intervals represent the 2.5% and 97.5% quantiles, respectively.

References

1. Dwyer-Lindgren, L., et al., *Mapping HIV prevalence in sub-Saharan Africa between 2000 and 2017.* Nature, 2019. **570**(7760): p. 189-193.

2. R Core Team, *R: A language and environment for statistical computing.* 2021, R Foundation for Statistical Computing: Vienna, Austria.

Supplementary Table 1: A comparison of three negative-binomial regression models that describe the expected probability of viral linkage between a pair of individuals randomly sampled from their respective communities in the BCPP trial. The models are fit ***(with and without) a transformation of the drive distance between communities***.

Variable	Coefficient	Standard Error	95% Conf. Interval	P value
Linear model				
Intercept	-11.31	0.54	-12.37 to -10.24	< 0.001
Transmission source: control community	0.90	0.51	-0.11 to 1.90	0.08
Transmission type: same community	2.05	0.61	0.86 to 3.25	0.001
Drive distance between communities in kilometers	-0.0031	0.0014	-0.0059 to -0.0003	0.03
	AIC	137.26		
	N	870		
Model with log transformed drive distance				
Intercept	-8.23	1.36	-10.90 to -5.57	< 0.001
Transmission source: control community	0.96	0.53	-0.08 to 2.01	0.07
Transmission type: same community	-1.04	1.41	-3.80 to 1.73	0.46
Log_e(drive distance between communities in kilometers)	-0.77	0.26	-1.28 to -0.25	0.004
	AIC	137.98		
	N	870		
Model with squared drive distance				
Intercept	-11.11	0.63	-12.35 to -9.88	< 0.001
Transmission source: control community	0.89	0.51	-0.11 to 1.90	0.08
Transmission type: same community	1.86	0.70	0.48 to 3.24	0.008
Drive distance between communities in kilometers	-0.0046	0.0033	-0.0110 to 0.0017	0.15
Squared (drive distance between communities in kilometers)	$1.70E-06$	$2.75E-06$	$-3.69E-06$ to $7.08E-06$	0.54
	AIC	139.03		
	N	870		

Notes:

1. All models were fit to directed opposite-sex HIV-1 transmission pairs (n = 31) identified between ordered pairs of communities in the BCPP trial during the post-baseline period (see methods section Deep-sequence phylogenetics data and supplementary note sections S1.1 and S1.2).
2. The linear model is the same as the post-baseline model in Table 1.
3. The reference category is directed opposite-sex HIV-1 transmission pairs where individuals were randomly sampled from different communities in the BCPP trial and the source of transmission resides in an intervention community.
4. The intercept denotes the risk of HIV-1 transmission (i.e. expected probability of viral linkage) in the reference category.
5. Coefficients, standard errors and confidence bounds are shown on the linear scale. Two-sided p-values are derived from the Wald Z-statistic (Z = Coefficient / Standard Error).
6. Transmission source: control community denotes the effect on the risk of HIV-1 transmission when the source of transmission resides in a control community.
7. Transmission type: same community denotes the effect on the risk of HIV-1 transmission when both the source and recipient reside within the same community.
8. Drive distance in the linear model denotes the effect on the risk of HIV-1 transmission for a 1 kilometer increase in the drive distance separating a pair of communities.
9. Drive distance in the log transformed distance model denotes the effect on the risk of HIV-1 transmission with a unit increase in the Log_e(drive distance) separating a pair of communities.
10. Drive distance in the squared distance model denotes the effect on the risk of HIV-1 transmission with a 1km increase and a 1km^2 increase in the drive distance separating a pair of communities.

Supplementary Table 2: Negative-binomial regression models describing the expected probability of viral linkage between a pair of individuals randomly sampled from their respective communities in the BCPP trial. Compared with Table 1, the models ***exclude potential partner co-visit events to clinics in intervention communities during baseline or post-baseline***.

Variable	Coefficient	Standard Error	95% Conf. Interval	P value
Baseline model: Before the intervention had taken effect				
Intercept	-11.44	0.51	-12.44 to -10.44	< 0.001
Transmission source: control community	0.57	0.31	-0.05 to 1.18	0.07
Transmission type: same community	3.46	0.50	2.48 to 4.43	< 0.001
Drive distance between communities in kilometers	-0.0026	0.0011	-0.0049 to -0.0004	0.02
	AIC	214.21		
	N	870		
Post baseline model: After the intervention had taken effect				
Intercept	-11.50	0.52	-12.51 to -10.48	< 0.001
Transmission source: control community	1.30	0.48	0.35 to 2.25	0.01
Transmission type: same community	2.09	0.63	0.86 to 3.32	0.001
Drive distance between communities in kilometers	-0.0038	0.0014	-0.0065 to -0.0011	0.01
	AIC	151.65		
	N	870		

Notes:

1. Negative binomial regression models were fit to directed opposite-sex HIV-1 transmission pairs identified between ordered pairs of communities in the BCPP trial at baseline (n = 49 / 51) and post-baseline (n = 27 / 31), excluding transmission pairs where both individuals visited a clinic within the same intervention community during the same period of the trial i.e. baseline or post-baseline (see supplementary note sections S1.1 and S1.2).
2. Post-baseline is described as at least one year after baseline household survey activities had concluded in a community.
3. The reference category is directed opposite-sex HIV-1 transmission pairs where individuals were randomly sampled from different communities in the BCPP trial and the source of transmission resides in an intervention community.
4. The intercept denotes the risk of HIV-1 transmission (i.e. expected probability of viral linkage) in the reference category.
5. Coefficients, standard errors and confidence bounds are shown on the linear scale. Two-sided p-values are derived from the Wald Z-statistic (Z = Coefficient / Standard Error).
6. Transmission source: control community denotes the effect on the risk of HIV-1 transmission when the source of transmission resides in a control community.
7. Transmission type: same community denotes the effect on the risk of HIV-1 transmission when both the source and recipient reside within the same community.
8. Drive distance between communities in kilometers denotes the effect on the risk of HIV-1 transmission for a 1 kilometer increase in the drive distance separating a pair of communities.

Supplementary Table 3: A comparison of the number of transmission pairs identified in the BCPP trial during the post-baseline period (n = 31) with those expected under the post-baseline model in Table 1. Table 1 describes the risk of HIV-1 transmission between communities.

Pairwise drive distance bin in kilometers	Median of bin	Number of distinct possible transmission pairs in bin	Number of identified transmission pairs	Number of expected transmission pairs
$[0, 50]$	25	226,500	19	26
$(50, 120]$	85	205,621	3	3
$(120, 220]$	170	209,215	5	3
$(220, 320]$	270	232,686	0	2
$(320, 410]$	365	256,466	2	2
$(410, 475]$	442.5	220,073	0	1
$(475, 710]$	592.5	197,104	1	1
$(710, 915]$	812.5	210,298	1	0
$(915, 1045]$	980	196,782	0	0
$(1045, 1200]$	1122.5	235,449	0	0

Notes:

1. The pairwise drive distances separating communities in the BCPP trial were grouped into ten bins that each had approximately similar numbers of the maximum number of distinct possible opposite-sex transmission pairs (see Model diagnostics in supplementary note section S1.2).
2. The expected number of transmission pairs was estimated with the post-baseline model in Table 1.
3. There was little evidence to suggest that the observed counts differed substantially from those expected under the post-baseline model in Table 1 (Fisher exact P = 0.792).

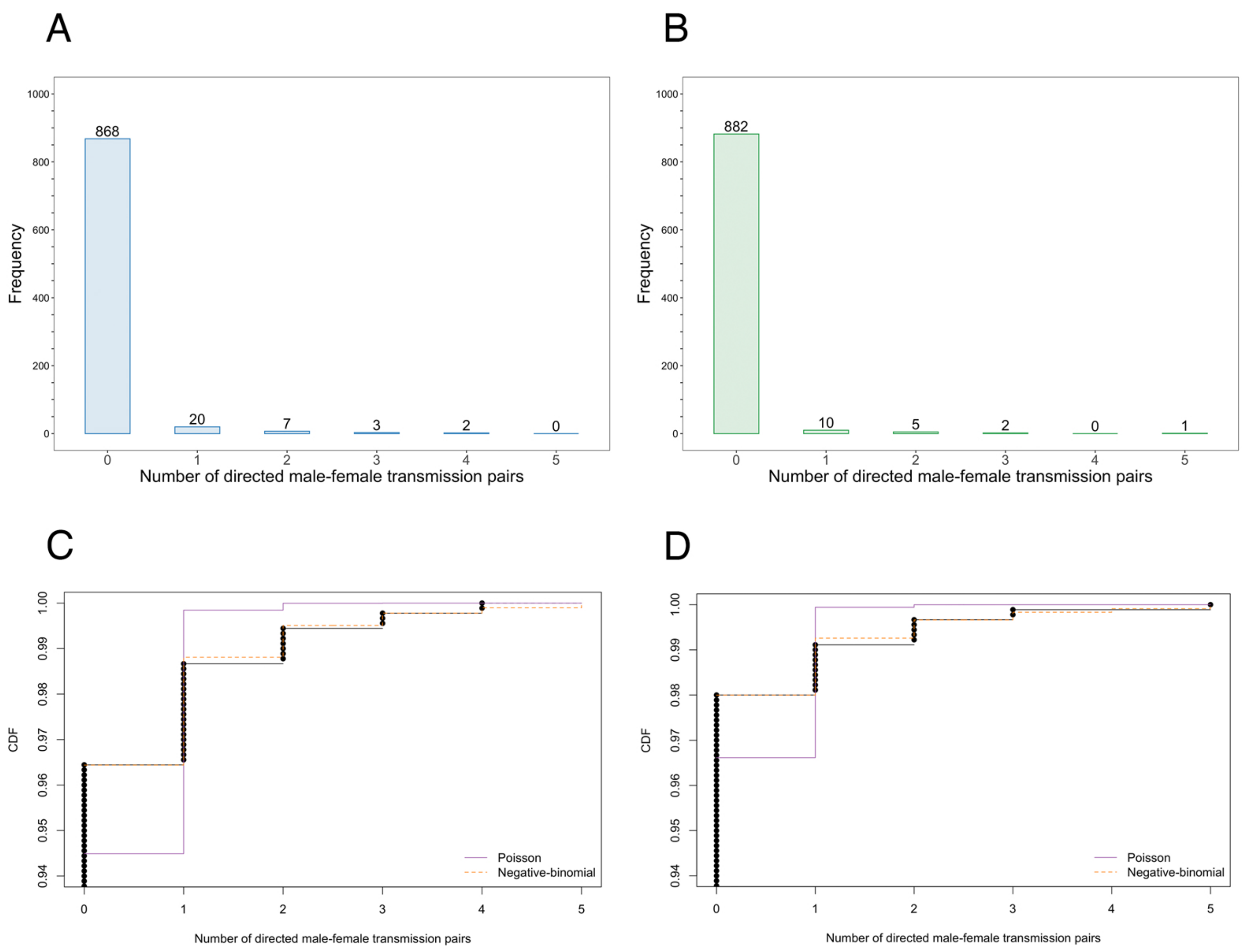

Supplementary Figure 1. Distribution of directed opposite-sex transmission pairs identified between ordered pairs of the 30 communities in the BCPP trial. The barplots in panels **A** and **B** show distributions of transmission pairs identified during the baseline (blue bars) and post-baseline (green bars) periods of the BCPP trial, respectively. For example, in panel A there were 20 ordered community pairs that each had a single identified opposite-sex transmission pair and 7 ordered community pairs that each had 2 identified transmission pairs. Panels **C** and **D** show the corresponding empirical and theoretical cumulative distribution functions (cdf) of identified transmission pairs during the baseline **(panel C)** and post-baseline **(panel D)** periods of the BCPP trial. In both panels **C** and **D** the empirical distribution is shown in black, and the theoretical Poisson and negative-binomial distributions are illustrated by solid purple lines and orange broken lines, respectively. The positively skewed distributions of identified transmission pairs in the BCPP trial are better approximated with a negative-binomial distribution compared to a Poisson distribution.

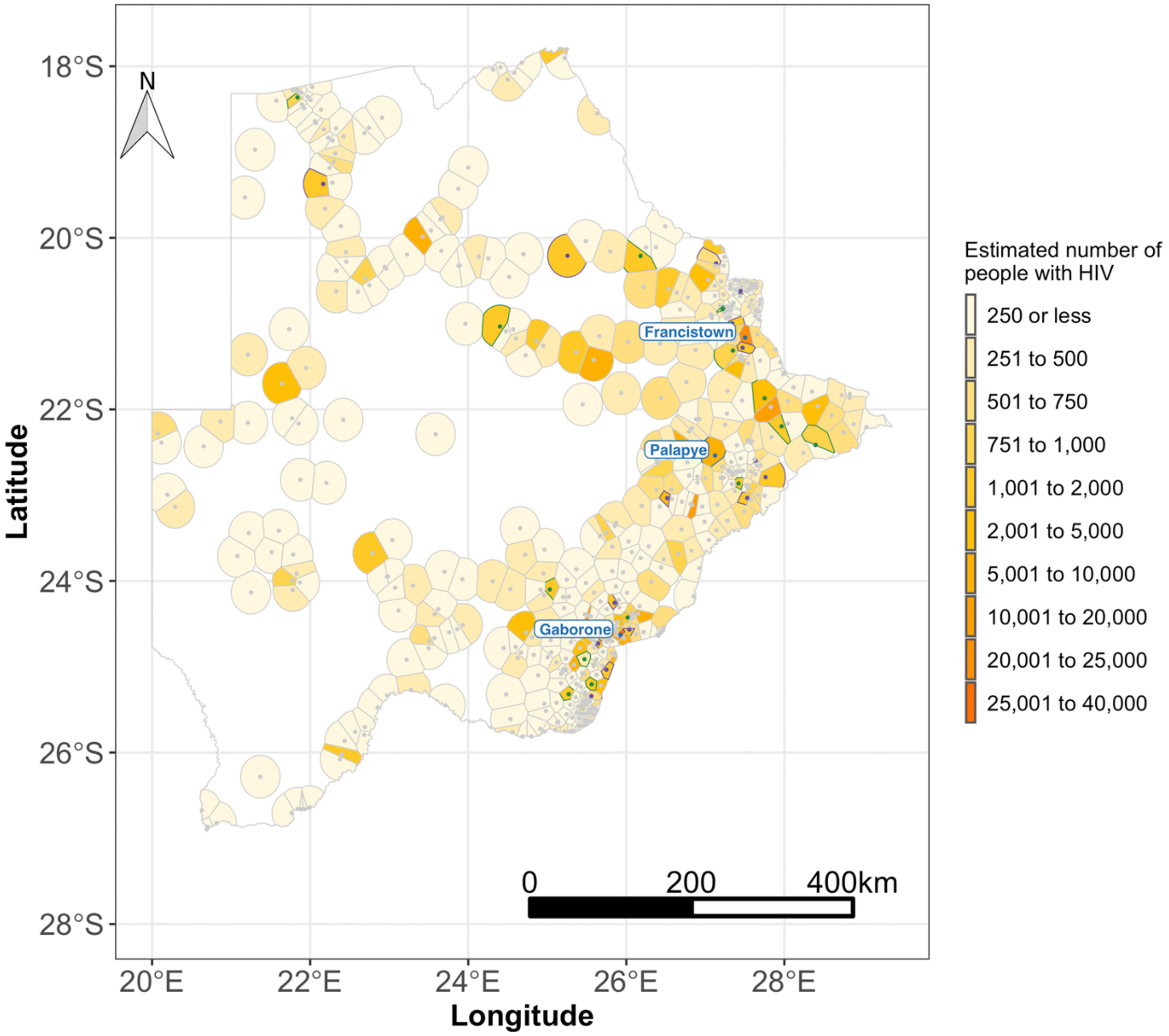

Supplementary Figure 2. Spatial distribution of the estimated number of people with HIV-1 in Botswana. Estimates of the number of people with HIV-1 were computed from district HIV-1 prevalence estimates from the 2013 Botswana AIDS Impact Survey (BAIS 2013) and community-size estimates from the 2011 Botswana population and housing census. Intervention communities in the BCPP trial are denoted by purple filled circles and boundaries and control communities are represented by green filled circles and boundaries. The communities in the BCPP trial are distributed around three major urban areas: Gaborone city, Palapye and Francistown city represented by blue filled circles and labels.

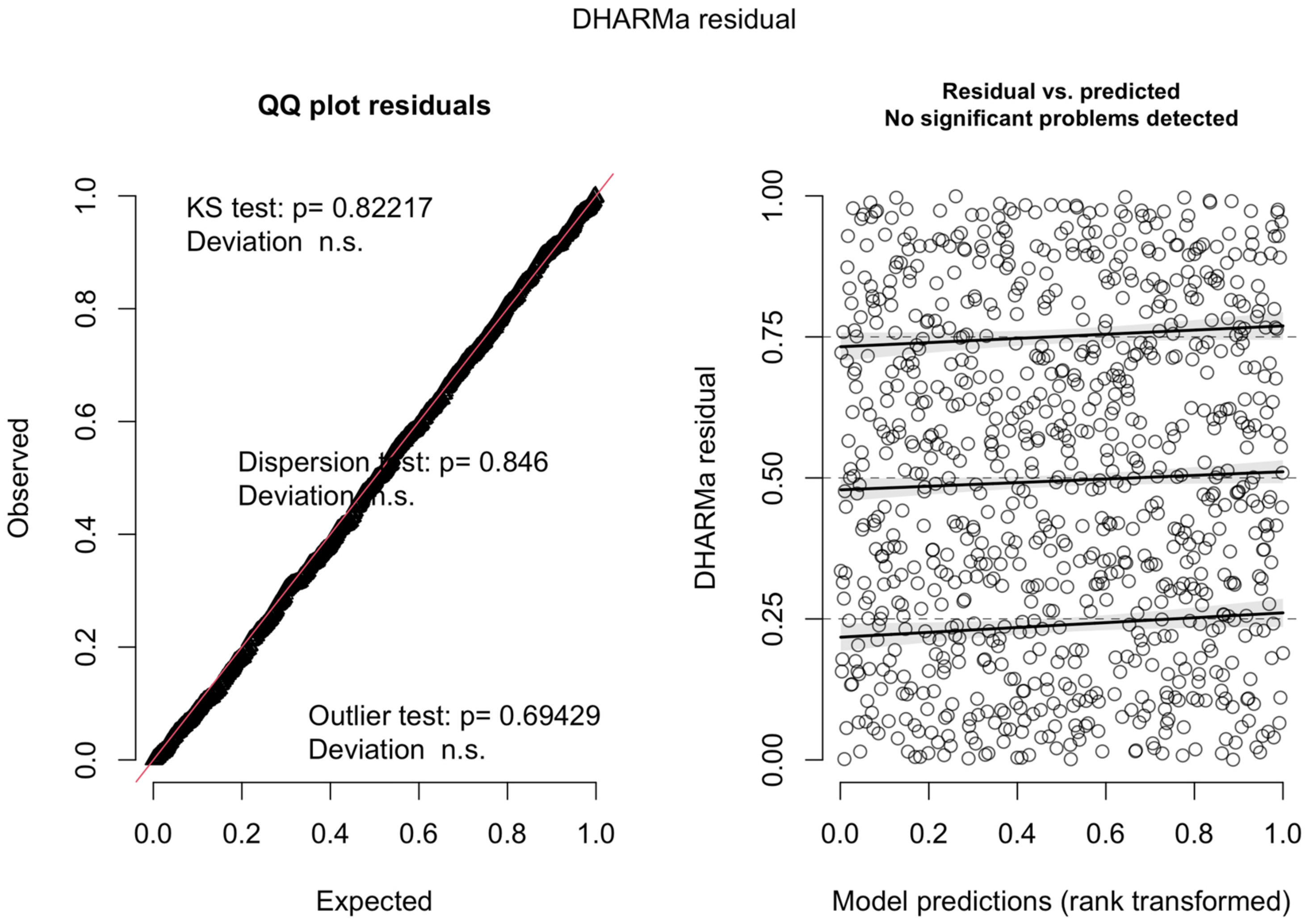

Supplementary Figure 3. A quantile-quantile (QQ) residual plot that compares the distribution of residuals of 31 opposite-sex transmission pairs identified between ordered pairs of communities in the BCPP trial during the post-baseline period with those that would be expected under the post-baseline model in Table 1.
