# Supplementary material for: Unpacking sources of transmission in HIV prevention trials with deep-sequence pathogen data
Source: Nat Commun. 2026 Mar 14;17:3935. doi: 10.1038/s41467-026-70203-x (PMC13128811; doi:10.1038/s41467-026-70203-x)
Supplement: Supplementary file 2 — Reporting Summary [file 41467_2026_70203_MOESM2_ESM.pdf]

nature portfolio

Corresponding author(s): Lerato E. Magosi, Marc Lipsitch

Last updated by author(s): Nov 16, 2025

Reporting Summary

Nature Portfolio wishes to improve the reproducibility of the work that we publish. This form provides structure for consistency and transparency in reporting. For further information on Nature Portfolio policies, see our Editorial Policies and the Editorial Policy Checklist.

Statistics

For all statistical analyses, confirm that the following items are present in the figure legend, table legend, main text, or Methods section.

n/a	Confirmed	
☐	☒	The exact sample size (*n*) for each experimental group/condition, given as a discrete number and unit of measurement
☐	☒	A statement on whether measurements were taken from distinct samples or whether the same sample was measured repeatedly
☐	☒	The statistical test(s) used AND whether they are one- or two-sided<br>*Only common tests should be described solely by name; describe more complex techniques in the Methods section.*
☐	☒	A description of all covariates tested
☐	☒	A description of any assumptions or corrections, such as tests of normality and adjustment for multiple comparisons
☐	☒	A full description of the statistical parameters including central tendency (e.g. means) or other basic estimates (e.g. regression coefficient) AND variation (e.g. standard deviation) or associated estimates of uncertainty (e.g. confidence intervals)
☐	☒	For null hypothesis testing, the test statistic (e.g. *F*, *t*, *r*) with confidence intervals, effect sizes, degrees of freedom and *P* value noted<br>*Give P values as exact values whenever suitable.*
☒	☐	For Bayesian analysis, information on the choice of priors and Markov chain Monte Carlo settings
☒	☐	For hierarchical and complex designs, identification of the appropriate level for tests and full reporting of outcomes
☒	☐	Estimates of effect sizes (e.g. Cohen's *d*, Pearson's *r*), indicating how they were calculated

Our web collection on statistics for biologists contains articles on many of the points above.

Software and code

Policy information about availability of computer code

Data collection	Publicly available pairwise drive distances between ordered pairs of 488 communities in the 2011 Botswana population and housing census were successfully sourced from the google distance matrix application programming interface (API) with the mapsapi package v0.5.0 in R v4.1.1 [Ref: 25].
Data analysis	mapsapi package v0.5.0 in R v4.1.1<br>nbreg module in Stata 13.1<br>glm.nb function in the MASS package v7.3-54 in R v4.1.2<br>predict function in the stats package in R v4.1.2<br>DHARMa package v0.4.6 in R v4.1.2<br><br>Code availability statement:<br>A code repository has been made available at the following URL: https://github.com/magosil86/spillover-infections [Ref: 26].<br>The code is also available via Zenodo at: https://doi.org/10.5281/zenodo.17641168.

For manuscripts utilizing custom algorithms or software that are central to the research but not yet described in published literature, software must be made available to editors and reviewers. We strongly encourage code deposition in a community repository (e.g. GitHub). See the Nature Portfolio guidelines for submitting code & software for further information.

Data

Policy information about availability of data

All manuscripts must include a data availability statement. This statement should provide the following information, where applicable:

- Accession codes, unique identifiers, or web links for publicly available datasets
- A description of any restrictions on data availability
- For clinical datasets or third party data, please ensure that the statement adheres to our policy

Data availability statement:
All relevant data are within the paper, figures and tables.

Research involving human participants, their data, or biological material

Policy information about studies with human participants or human data. See also policy information about sex, gender (identity/presentation), and sexual orientation and race, ethnicity and racism.

Reporting on sex and gender	Of the 82 directed opposite-sex transmission pairs identified in the BCPP study, there were 51 (21 female-to-male, 30 male-to-female) baseline pairs and 31 (16 female-to-male, 15 male-to-female) post-baseline pairs [Refs: 1, 9]. Sex and age were self-reported [Refs: 1, 9].
Reporting on race, ethnicity, or other socially relevant groupings	The BCPP study recruited participants with documented Botswana citizenship or marriage to a citizen of Botswana [Ref: 1].
Population characteristics	BCPP study description. The Botswana Combination Prevention Project (BCPP, also known as the Ya Tsie trial) was a pair-matched community-randomized trial to evaluate the effect of universal HIV testing and treatment on HIV incidence reduction. The trial was conducted from 2013-2018 in 30 rural and peri-urban communities distributed across Botswana [Refs: 1, 20]. The BCPP intervention was administered through an existing system of highly decentralized health care facilities and personnel overseen by District Health Management Teams (DHMT's) of health care workers in the Ministry of Health.
Recruitment	Trial participants were adults aged 16-64 years and the average population size eligible to participate in each trial community was 3,820 people. Communities were matched into 15 pairs based on three criteria: geographical proximity to major urban areas (Gaborone city, Palapye and Francistown city), population size and age structure, and access to health services; then within each pair, communities were randomized into the intervention and control arms of the trial [Refs: 1, 9].<br><br>Trial participants with HIV were invited to provide a sample for viral phylogenetic analysis. This included all people with HIV from (1) the baseline household survey, (2) annual household surveys, (3) end-of-study survey, as well as (4) all people with HIV (but not yet on ART) who were referred for treatment during community-wide testing and counseling campaigns, (5) all people with HIV that later presented at health care facilities in intervention communities and (6) all people with HIV who were already receiving HIV care at health facilities in intervention communities [Refs: 1, 9].
Ethics oversight	Ethics statement. The BCPP study was approved by the Botswana Health Research and Development Committee and the institutional review board of the Centers for Disease Control and Prevention; and was monitored by a data and safety monitoring board and Westat. Written informed consent for enrollment in the study and viral HIV genotyping was obtained from all participants.

Note that full information on the approval of the study protocol must also be provided in the manuscript.

Field-specific reporting

Please select the one below that is the best fit for your research. If you are not sure, read the appropriate sections before making your selection.

☐ Life sciences ☐ Behavioural & social sciences ☒ Ecological, evolutionary & environmental sciences

For a reference copy of the document with all sections, see nature.com/documents/nr-reporting-summary-flat.pdf

Ecological, evolutionary & environmental sciences study design

All studies must disclose on these points even when the disclosure is negative.

Study description	This study is an analysis of data collected during the Botswana Combination Prevention Project (BCPP) [Refs 1, 9]. Publicly available counts of 82 directed opposite-sex transmission pairs between ordered pairs of the 30 communities in the BCPP study were sourced from [Ref: 9] and were used to estimate the relative contribution of different sources of infection in the BCPP study.
Research sample	Counts of 82 directed opposite-sex transmission pairs from the BCPP study.
Sampling strategy	All available opposite-sex transmission pairs from the BCPP study were used.

Data collection	Publicly available counts of 82 directed opposite-sex transmission pairs between ordered pairs of the 30 communities in the BCPP study were sourced from [Ref: 9]
Timing and spatial scale	All 82 directed opposite-sex transmission pairs in the BCPP study were sampled in Botswana between 2013 and 2018.
Data exclusions	All 82 directed opposite-sex transmission pairs identified in the BCPP study were used (no exclusions).
Reproducibility	We used opposite-sex transmission pairs from HIV viral genomes circulating in Botswana, that is, a natural experiment. Analysis can be reproduced using the Github code repository at the following URL: https://github.com/magosil86/spillover-infections [Ref: 26].
Randomization	We used opposite-sex transmission pairs from HIV viral genomes circulating in Botswana, that is, a natural experiment.
Blinding	We used opposite-sex transmission pairs from HIV viral genomes circulating in Botswana, that is, a natural experiment.

Did the study involve field work? ☐ Yes ☒ No

Reporting for specific materials, systems and methods

We require information from authors about some types of materials, experimental systems and methods used in many studies. Here, indicate whether each material, system or method listed is relevant to your study. If you are not sure if a list item applies to your research, read the appropriate section before selecting a response.

Materials & experimental systems

n/a	Involved in the study	
☒	☐	Antibodies
☒	☐	Eukaryotic cell lines
☒	☐	Palaeontology and archaeology
☒	☐	Animals and other organisms
☒	☐	Clinical data
☒	☐	Dual use research of concern
☒	☐	Plants

Methods

n/a	Involved in the study	
☒	☐	ChIP-seq
☒	☐	Flow cytometry
☒	☐	MRI-based neuroimaging

Plants

Seed stocks	No seed stocks were used in this study.
Novel plant genotypes	No plants were used in this study.
Authentication	No seed stocks or plants were used in this study.
